# Interventional human ocular safety experiments for 222‐nm far‐ultraviolet‐C lamp irradiation

**DOI:** 10.1111/php.14016

**Published:** 2024-08-19

**Authors:** Kazunobu Sugihara, Sachiko Kaidzu, Masahiro Sasaki, Masaki Tanito

**Affiliations:** ^1^ Department of Ophthalmology Shimane University Faculty of Medicine Izumo Japan; ^2^ Ushio Inc Tokyo Japan

**Keywords:** 222‐nm excimer lamp, erythema, health hazard, photokeratitis, ultraviolet germicidal irradiation (UVGI)

## Abstract

The study aimed to directly assess the ocular safety of 222‐nm far‐ultraviolet‐C (UVC) irradiation in humans, given the limited clinical trials in this area. This wavelength offers the potential for safe and effective microbial inactivation in occupied spaces, but its safety profile for human eyes requires thorough investigation. This prospective, interventional study involved five subjects aged 29–47 years, who were exposed to 222‐nm UVC at doses of 22, 50, and 75 mJ/cm^2^. The subjects were monitored using custom‐made glasses with a UV‐cut filter on one eye to serve as a control. UVC irradiation was conducted using a KrCl excimer lamp, and ocular examinations were performed prior to exposure, 24 h post‐exposure, and at 1, 3, and 6 months. Parameters assessed included visual acuity, refractive error, corneal endothelial density, corneal erosion scores, and conjunctival hyperemia scores. The study found no clinically significant photokeratitis or long‐term eye damage across the five subjects, even at the highest dose of 75 mJ/cm^2^. Temporary ocular discomfort, including sensations of dryness and epiphora, was reported, but these symptoms subsided within hours after irradiation. The findings indicate that 222‐nm far‐UVC irradiation up to 75 mJ/cm^2^ does not cause “clinically significant photokeratitis” or long‐term ocular damage, though it may induce temporary discomfort. This supports the safe use of 222‐nm UVC for germicidal applications in occupied environments, providing a basis for revised safety guidelines.

## Graphical abstract

To directly assess the ocular safety of 222‐nm far‐ultraviolet‐C (UVC) irradiation in humans, five subjects were exposed to 222‐nm UVC at doses of 22, 50, and 75 mJ/cm^2^. The findings indicate that far‐UVC irradiation does not cause “clinically significant photokeratitis” or long‐term ocular damage, though it may induce temporary discomfort.

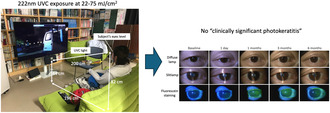

**Abbreviations**
ACGIH: The American Conference of Governmental Industrial Hygienists
BCVA: best‐corrected visual acuity
CECD: corneal endothelial cell density
KrBr: krypton‐bromide
KrCl: krypton‐chloride
SERE: spherical equivalent refractive error
SPK: superficial punctate keratopathy
TLV: threshold limit value
UV: ultraviolet
UVGI: ultraviolet germicidal irradiation

## INTRODUCTION

Ultraviolet (UV) radiation spans wavelengths from 100 to 400 nm, categorized into UVC (100–280 nm), UV‐B (280–315 nm), and UV‐A (315–400 nm). Previous research has indicated that exposure to longer UV wavelengths, such as UV‐B and UV‐A, can cause biological effects like photokeratitis and corneal opacity,^1^ as well as chronic conditions such as pterygium, droplet keratopathies, and cortical cataract.^2^, ^3^, ^4^ While UVC has a potent germicidal effect on viruses and bacteria, its traditional use in human‐occupied spaces is limited to unoccupied areas like the upper room air to avoid direct exposure, which could potentially cause skin erythema and photokeratitis.^5^, ^6^

Recently, shorter‐wavelength ultraviolet germicidal irradiation (UVGI) sources, such as krypton‐bromide (KrBr) and krypton‐chloride (KrCl) excimer lamps emitting at 207 and 222 nm, respectively, have gained attention for their ability to provide full‐room irradiation that is considered safe for humans while effectively inactivating microbes.^7^, ^8^, ^9^, ^10^ Animal studies have demonstrated that the UV penetration depth in the rat corneal epithelium strongly depends on the wavelength. Far‐UVC wavelengths like 207 and 222 nm only penetrate to the superficial cellular layers of the corneal epithelium, which are shed within approximately 24 h due to the natural turnover cycle.^11^ This minimal penetration and rapid turnover are likely reasons for the reduced hazard of Far‐UVC.^12^, ^13^, ^14^ A 1‐year prospective observation in a hospital outpatient clinic showed that a full‐room UVGI setting with 222‐nm lamp units caused no acute or chronic health effects in subjects while effectively inactivating bacteria and phages in the room.^15^

The American Conference of Governmental Industrial Hygienists (ACGIH) previously set the threshold limit value (TLV) for 222 nm at 22 mJ/cm^2^ per day,^5^ which has now been revised to 160 mJ/cm^2^ per day.^10^, ^16^, ^17^ A 50‐year‐old study determined photokeratitis thresholds for 215–225 nm as 46 mJ/cm^2^ in rabbits, 21 mJ/cm^2^ in primates, and 10 mJ/cm^2^ in humans.^18^ Recent research using rat models and high‐throughput KrBr and KrCl excimer lamps found that the minimal photokeratitis threshold doses for 207 and 222 nm were 15,000 and 5000 mJ/cm^2^, respectively.^13^ Consequently, the ACGIH's TLV is much lower than the photokeratitis level found in rat experiments but higher than the dose identified in earlier human studies. In pathogen‐contaminated environments, a minimum dose of 27 mJ/cm^2^ of 222‐nm UVC was necessary for over 95% germicidal activity against gram‐negative and gram‐positive bacteria. A dose of 25.1 mJ/cm^2^ was sufficient for more than 95% virucidal activity against low‐pathogenic avian influenza virus and SARS‐CoV‐2.^19^ This study was conducted to confirm the eye safety of far‐UVC doses at levels required for UVGI in humans.

## MATERIALS AND METHODS

### Study designs and subjects

This prospective, interventional study was conducted at the Department of Ophthalmology, Shimane University Faculty of Medicine. The study adhered to the tenets of the Declaration of Helsinki and Ethical Guidelines for Medical and Health Research Involving Human Subjects in Japan. Prior to the start of study, the protocol was reviewed and approved by the Institutional Review Board of Shimane University Hospital (IRB No.: 20201130‐1, approval date December 28, 2020) and registered at the public clinical trial registration site (UMIN000042867). All the subjects provided written informed consent for participation in the study. The inclusion criteria that need to be fulfilled were: an age range of 20–49 years old; the person who can avoid excessive UV exposure (such as outdoor sports, sea swimming, and climbing) within 48 h preceding the experimental UV irradiation and scheduled follow‐up visits; decimal best‐corrected visual acuities of 0.9 or better in both eyes, and uncorrected visual acuity by both eyes of 0.3 or better (to ensure the possibility to watch movies during UV irradiation); no ocular pathologies that affected visual acuity; and no use of topical eye or facial skin medications. The exclusion criteria were contact lens wearer, engaged in occupations that exposed the subject to UV radiation on a daily basis (e.g., outdoor work and welding), a corneal erosion density score of 2 or higher or a conjunctival hyperemia score of 2 or higher prior to the UV irradiation; dry eye disease, atopic dermatitis, sunlight sensitivity, and current participants of other clinical trials. As a result, this study included five subjects (age range, 29–47 years; mean ± SD age of 40.0 ± 7.3 years; 4 males) (Table 1).

**TABLE 1:** Summary of subjects.

Subject ID	UVC01	UVC02	UVC03	UVC04	UVC05
Age	37	46	29	47	41
Gender	Female	Male	Male	Male	Male

	Right eye	Left eye	Right eye	Left eye	Right eye	Left eye	Right eye	Left eye	Right eye	Left eye
Total irradiation dose, mJ/cm^2^	22	0	50	0	50	0	75	0	75	0
Irradiance, μW/cm^2^	3.1–3.4	0	3.1–3.4	0	3.1–3.4	0	3.1–3.4	0	3.1–3.4	0
Irradiation time, h	2	2	4	4	4	4	6	6	6	6

### Settings of UVC irradiation

**FIGURE 1:**
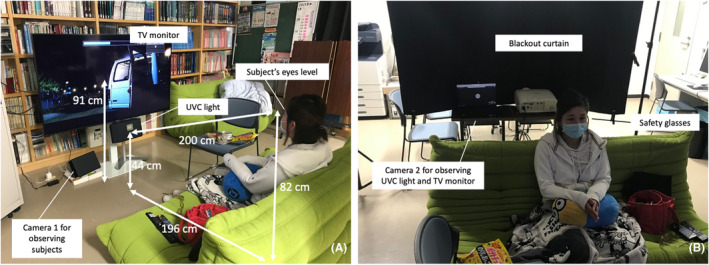
Settings of ultraviolet‐C (UVC) irradiation. (A) Device setting in front of the subject. (B) Devices setting behind the subject.

**FIGURE 2:**
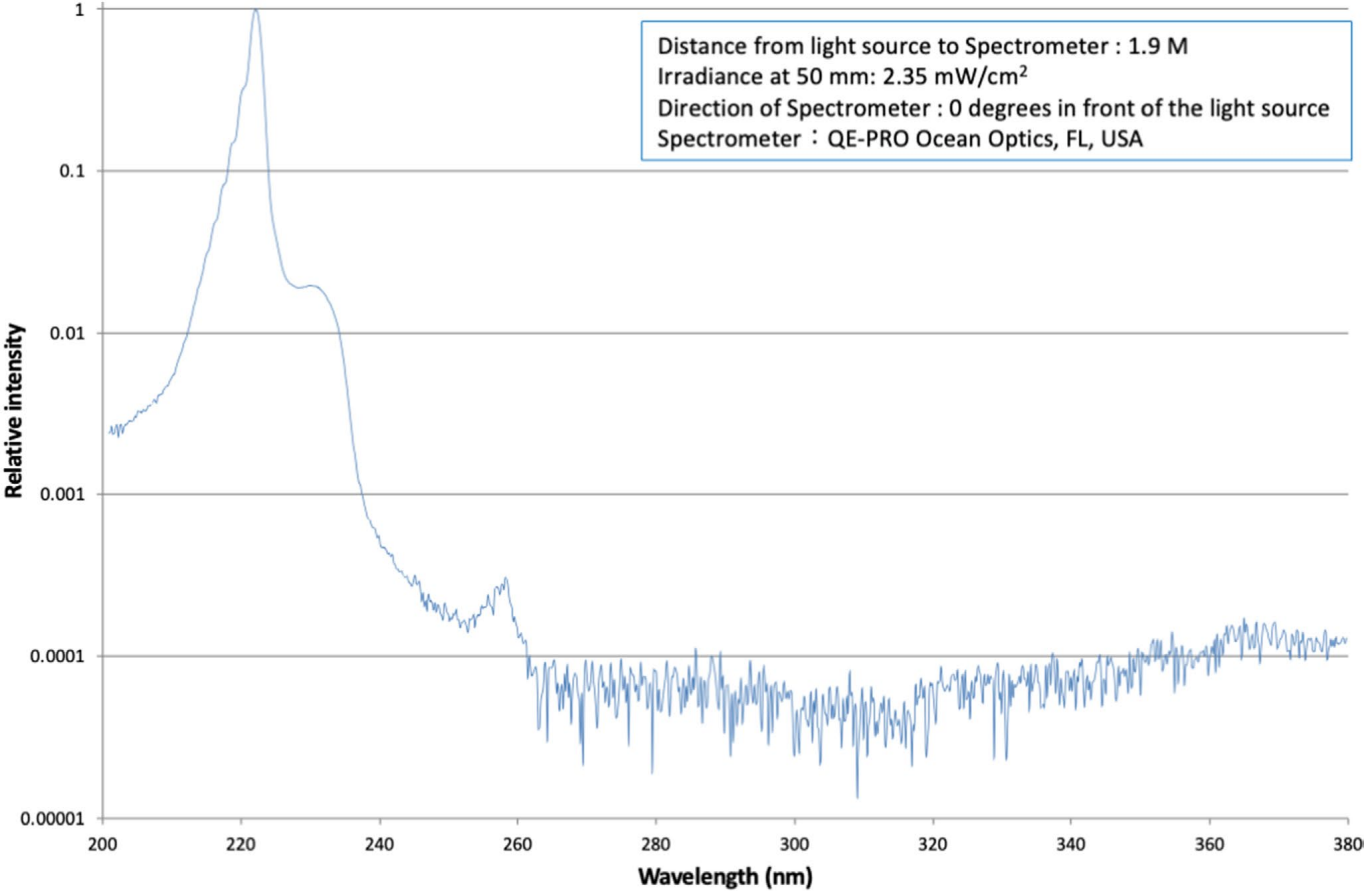
Spectral distribution of the 222‐nm far‐ultraviolet‐C (UVC) lamps used in this study. The vertical axis of the data is shown in Log_10_ unit.

**FIGURE 3:**
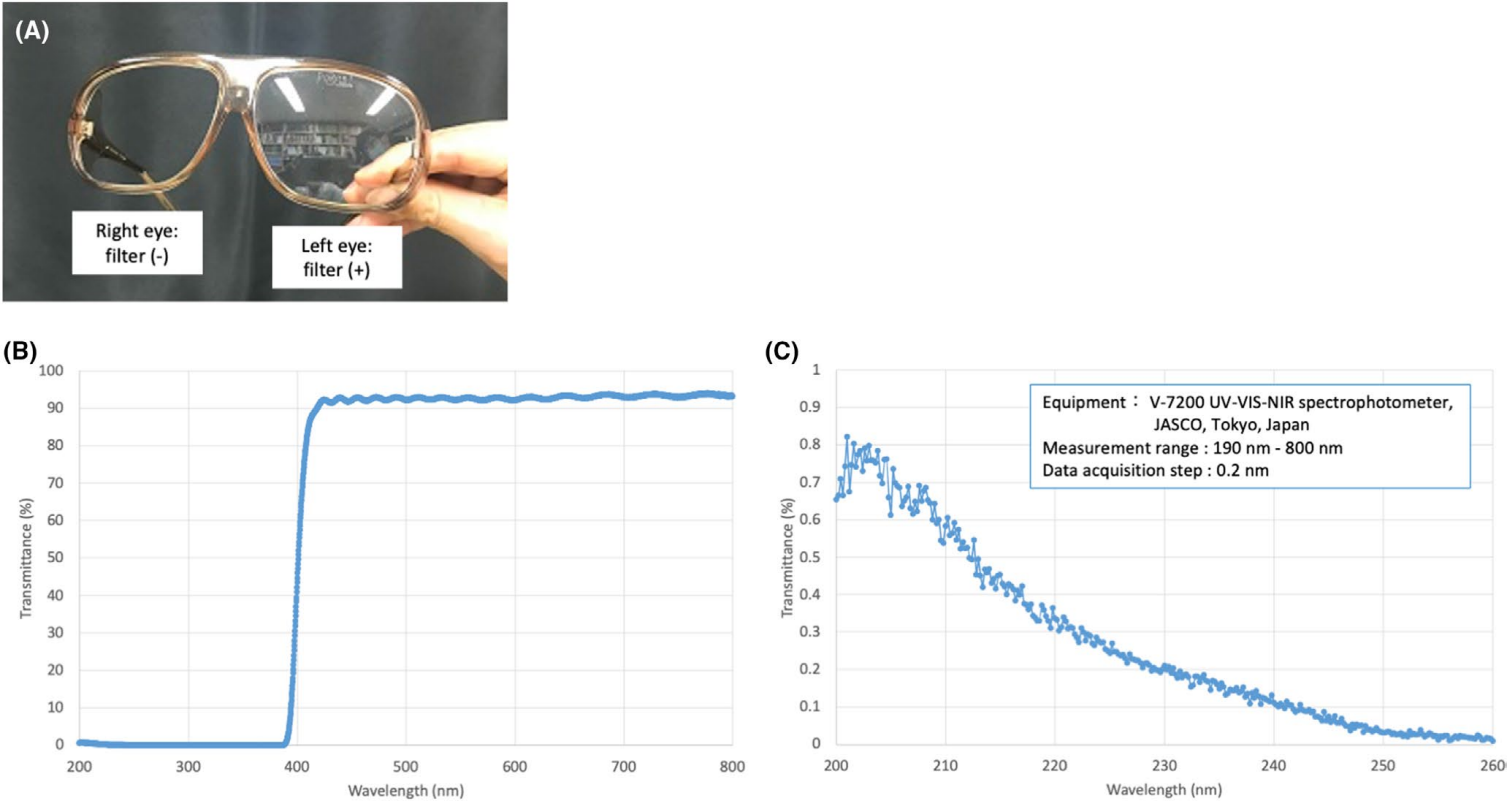
Custom‐made glasses used in this study. (A) Appearance of the glasses. (B) Transmittance of the filter on the left side of the glasses with a maximum *y*‐axis value of 100%. (C) Transmittance of the filter on the left side of the glasses with a maximum *y*‐axis value of 1%.

The setting of the UVC irradiation is shown in Figure 1A,B. The TV monitor and sofa were placed so that the distance between the surface of the monitor and the back of the sofa was 196 cm. A mercury‐free, KrCl excimer UVC lamp unit (Care222 model U3‐Z2, USHIO Inc., Tokyo, Japan) was placed under the TV monitor and adjusted so that the distance between the subject's eyes and the UVC lamp was 200 cm. One camera unit was placed in front of the subject (Camera 1) and one behind the subject (Camera 2) to monitor the irradiation process. The spectral distribution measured by a spectrometer (QE‐PRO Ocean Optics, FL, USA) is shown in Figure 2. Each unit emitted a peak wavelength of 222 nm and had a cut‐off filter that cut wavelengths longer than 230 nm, and totally cut wavelength longer than 240 nm. The impurity of light wavelength (235–320 nm/200–230 nm) was calculated to be 0.55% for this unit. The housing of the lamp unit was covered by black paper, installed at a height of 40 cm above the floor, and directed to the subject's eye level (Figure 1A). During UVC irradiation, the subjects wore custom‐made glasses with no filter on the right side and a UV‐cut filter (YS180, Yamamoto Kogaku, Higashi‐Osaka, Japan) on the left side (Figure 3A). The spectral transmittance of the left side filter measured by a V‐7200 UV–VIS–NIR spectrophotometer (JASCO, Tokyo, Japan) was shown in Figure 3B,C. The filter cut wavelengths shorter than 400 nm, and the transmittance was around 0.3% at 222 nm. The filter transmissions appear to increase at lower wavelengths, albeit only slightly. This may be detector noise, as it appears to reflect the reciprocal of the UV photocathode spectral response. Therefore, the right eyes were the irradiated eyes, and the left eyes were the non‐irradiated controls in this study.

**FIGURE 4:**
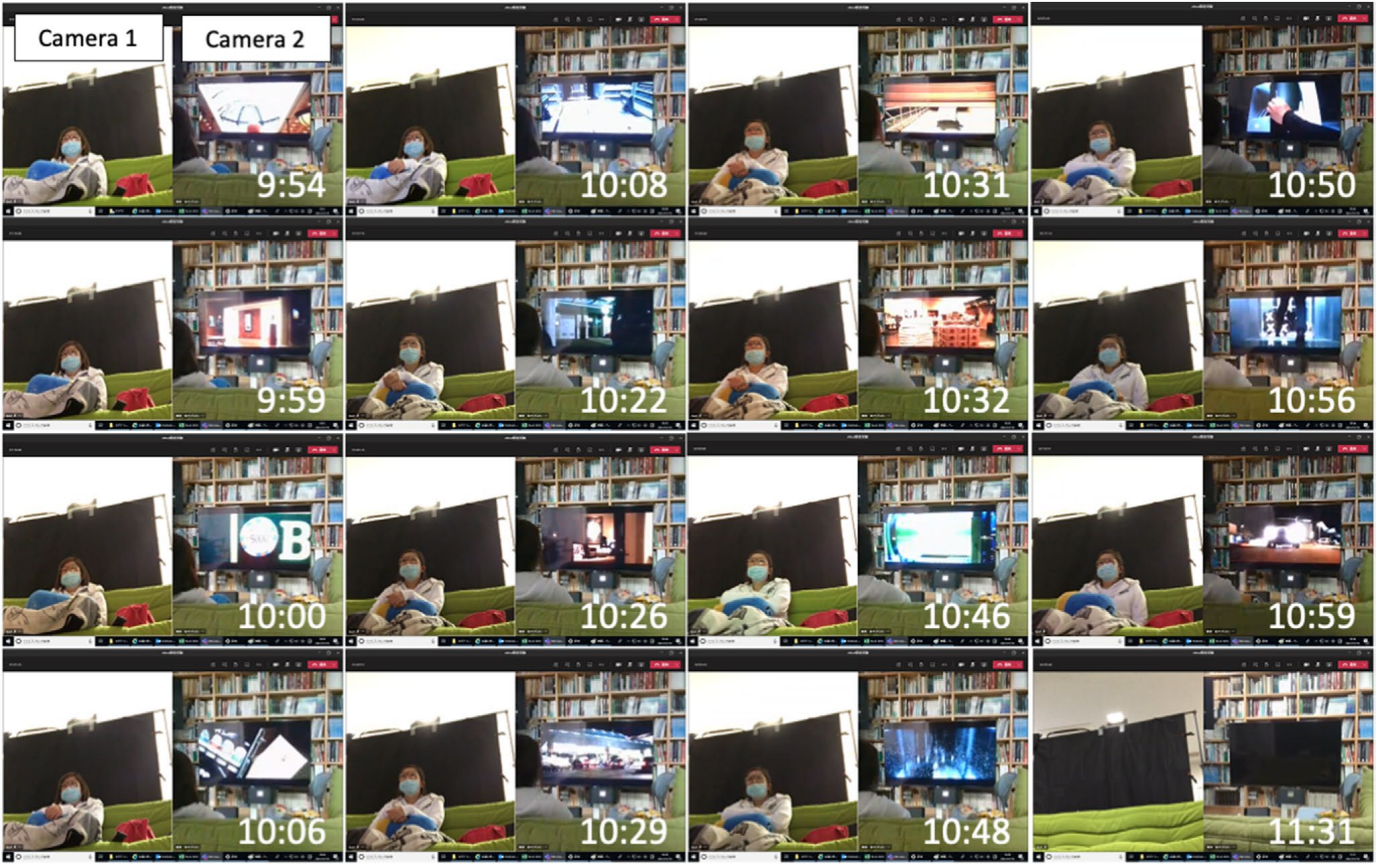
Typical monitoring images during irradiation (UVC01).

The right eyes of subjects were irradiated to achieve total irradiation doses of: 22 mJ/cm^2^ (*n* = 1, study ID = UVC01), 50 mJ/cm^2^ (*n* = 2, UVC02 and UVC03), or 75 mJ/cm^2^ (*n* = 2, UVC04 and UVC05) (Table 1). The different irradiation doses were achieved by the changes in irradiation duration (2‐, 4‐, and 6‐h irradiation for 22, 50, and 75 mJ/cm^2^ irradiation doses, respectively) at the same irradiance (3.1–3.4 μW/cm^2^). Prior to the irradiation, the irradiance was measured at the subject's eye level behind the transmitting spectacle lens with an ultraviolet irradiance meter (VUV‐S172/UIT‐250 USHIO INC) by directing the detector to the lamp. During the irradiation, the subjects spent time watching a movie on a TV monitor (91 cm high at the center of the monitor). The subjects were monitored on another TV monitor in a separate room during irradiation using images sent from Cameras 1 and 2 to confirm that they were not dozing or looking away, and to warn them when necessary to view the screen (Figure 4). For the 50 and 75 mJ/cm^2^ irradiations, a 10‐min break was taken after every 2 h of irradiation.

### Scheduled examinations

**FIGURE 5:**
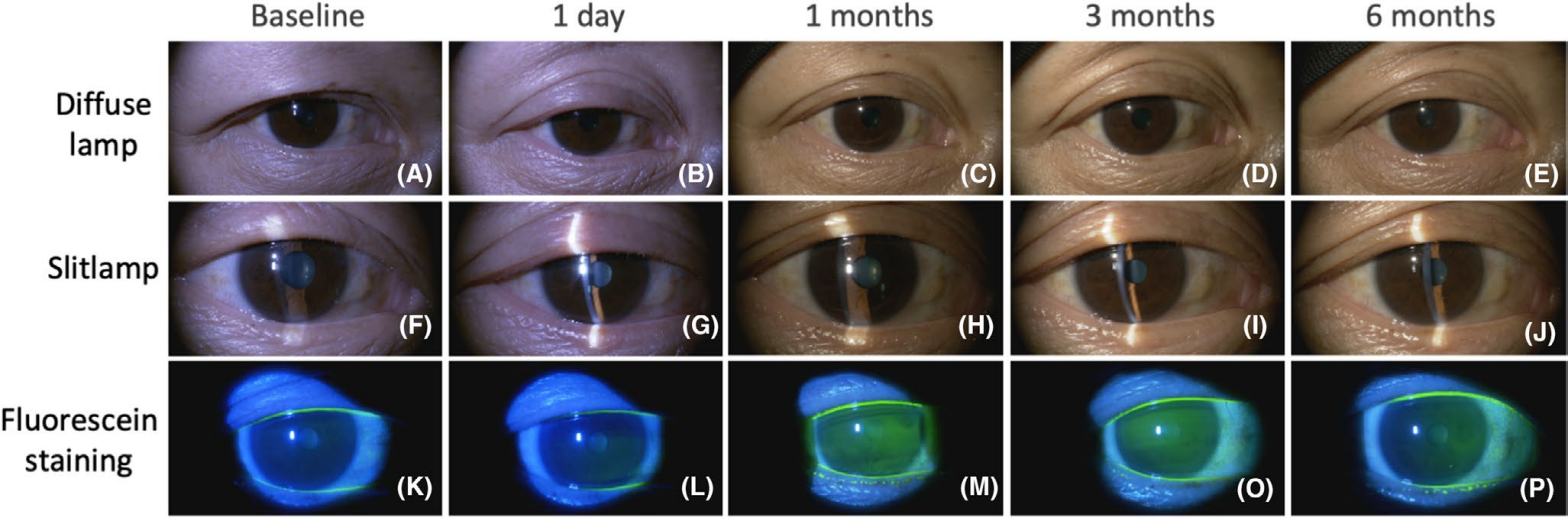
Representative slit lamp photographs of 75 mJ/cm^2^ irradiated eye (UVC05, right eye). Conditions for observation of lid skin change (A–E), conjunctival hyperemia, pterygium, cataract (F–J), and corneal erosion (K–P).

For the acquisition of ocular safety profiles, the participants were examined within 1 week prior to the irradiation (for baselines), at 24 h after the start of irradiation (for 1 day), and at 1, 3, and 6 months after the irradiation. In each examination, bilateral best‐corrected visual acuity (BCVA), spherical equivalent refractive error (SERE), slit lamp examination findings, corneal endothelial cell density (CECD), subjective symptoms, and any other potentially adverse events were recorded. Measurements of SERE and CECD were omitted in the first‐day examination. Visual acuity was measured using a decimal visual acuity chart. SERE was measured by using an autorefract‐keratometer (TonoRef III, Nidek, Gamagori, Japan). A slit lamp examination using a slit lamp microscope 4ZL (Takagi, Nagano, Japan) was used to obtain a corneal erosion score, conjunctival hyperemia score, and presence or absence of pterygium or cataract. The slit lamp microscope was also used to record any potential lid skin changes (erythema, increase or decrease in pigmentation). Lid skin change was assessed with 6.3× magnification (Figure 5A–E), and the other signs were assessed with 10× magnification (Figure 5F–P). For assessment of corneal erosion, the ocular surface was stained with a sodium‐fluorescein solution for observation under a blue light (Figure 5K–P). For assessment of cataract, both thin slit‐beam and diffuse lights were used for observation (Figure 5F–J), while the other examinations were observed with diffuse light (Figure 5A–E,K–P). Corneal erosion was scored in each area (0–3; 0, no punctate staining; 1, staining area occupied less than one third of the cornea; 2, staining area occupied one to two thirds of the cornea; and 3, the staining area occupied greater than two thirds of the cornea.) and the presence (density, 0–3; 0, no staining; 1, sparse density; 2, moderate density; and 3, high density and overlapped lesion by reference to standard photographs) of superficial punctate keratopathy (SPK).^20^ Scoring of conjunctival hyperemia was determined based on the Japanese Guidelines for Allergic Conjunctival Disease 2020; where a score 0 = no manifestation, a score 1 = dilatation of several vessels, a score 2 = dilation of many vessels, and a score 3 = impossible to distinguish individual blood vessels.^21^ CECD was measured using a specular microscope (EM‐3000; Tomey Corporation, Nagoya, Japan). All the examinations/measurements were performed by an experienced ophthalmologist (K.S.) and orthoptists. To ensure the safety of the subjects, exposures were delivered stepwise, such that the 50 mJ/cm^2^ irradiation was performed only after confirming that no significant ocular damage (i.e., corneal erosion density score of 3) causally related to the irradiation had occurred by 1 month following the 22 mJ/cm^2^ irradiation. Similarly, a 75 mJ/cm^2^ irradiation was only performed after confirming that no significant ocular damage causally related to the irradiation had occurred 3 months after the 22 mJ/cm^2^ irradiation or 1 month after the 50 mJ/cm^2^ irradiation.

## RESULTS

The results are summarized in Table 2. During the follow‐up period from 1 day to 6 months after the irradiation, the safety indices including VA, SERE, corneal erosion area and density scores, conjunctival hyperemia score, and CECD did not change for both eyes of the five subjects. During the same period, pterygium, cataract and face skin change were not detected for any of the five subjects. Subject‐reported events within 24 h of the start of irradiation, as reported in interviews during Day 1 observations, were summarized in Table 3. From the subjects, epiphora sensation, dry eye sensation, epiphora, eye discomfort, conjunctival hyperemia, and mild pain were reported. All reports were for the right eye, and these events started 2–2.5 h after the start of irradiation and disappeared 4.5–11 h after the start of irradiation. No events were reported for the left eye.

**TABLE 2:** Summary of results.

Subject ID	UVC01		UVC02		UVC03		UVC04		UVC05	
	Right eye (22 mJ/cm^2^)	Left eye (control)	Right eye (50 mJ/cm^2^)	Left eye (control)	Right eye (50 mJ/cm^2^)	Left eye (control)	Right eye (75 mJ/cm^2^)	Left eye (control)	Right eye (75 mJ/cm^2^)	Left eye (control)
Corrected VA, decimal										
Baseline	1.2	1.2	1.2	1.2	1.2	1.2	1.5	1.5	1.5	2
1M	1.2	1.2	1.2	1.2	1.2	1.2	1.2	1.2	1.5	1.5
3M	1.2	1.2	1.5	1.5	2	1.5	1.2	1.2	1.2	1.2
6M	1.2	1.2	1.2	1.2	1.2	1.2	1.5	1.5	1.5	1.5
SERE, D										
Baseline	−3.625	−4.25	0	−0.125	+0.125	+0.125	−0.625	−0.625	+0.125	−0.125
1M	−3	−3.5	−0.25	−0.25	+0.125	+0.125	−0.5	−0.625	+0.125	+0.125
3M	−3	−3.375	+0.125	+0.125	+0.125	0	−0.25	−0.25	+0.125	0
6M	−3.875	−4	0	+0.25	−0.125	0	−0.125	0	+0.375	0
Corneal erosion area score										
Baseline	1	1	0	0	1	1	0	0	0	1
1D	1	1	0	0	1	1	1	0	1	1
1M	1	2	0	0	1	1	0	0	1	1
3M	0	0	0	0	0	0	0	0	0	0
6M	0	0	0	0	1	1	0	0	0	1
Corneal erosion density score										
Baseline	1	1	0	0	1	1	0	0	0	1
1D	1	1	0	0	1	1	1	0	1	1
1M	1	1	0	0	1	1	0	0	1	1
3M	0	0	0	0	0	0	0	0	0	0
6M	0	0	0	0	1	1	0	0	0	1
Conjunctival hyperemia score										
Baseline	0	0	0	0	0	0	0	0	0	0
1D	0	0	0	0	0	0	0	0	0	0
1M	0	0	0	0	0	0	0	0	0	0
3M	0	0	0	0	0	0	0	0	0	0
6M	0	0	0	0	0	0	0	0	0	0
CECD, cells/cm^2^										
Baseline	2733	2642	2967	2974	2586	2737	2726	2748	2681	2608
1M	2701	2880	2799	2791	2668	2602	2892	2811	2701	2487
3M	3050	2916	3006	2819	2700	2780	2651	2624	2823	2745
6M	2670	2881	2987	2849	2783	2732	2806	2740	2814	2625
Pterygium	NR	NR	NR	NR	NR	NR	NR	NR	NR	NR
Cataract	NR	NR	NR	NR	NR	NR	NR	NR	NR	NR
Face skin change	NR	NR	NR	NR	NR	NR	NR	NR	NR	NR

Abbreviations: CECD, corneal endothelial cell density; NR, not reported; SERE, spherical equivalent refractive error.

**TABLE 3:** Subject‐reported events within 24 h after the start of irradiation.

	Events	Events started from the start of irradiation	Events lasted from the start of irradiation
UVC01, right eye (22 mJ/cm^2^)	Epiphora sensation	2.5 h	4.5 h
UVC02, right eye (50 mJ/cm^2^)	Dry eye sensation, mild photophobia, and epiphora sensation	2 h	7 h
UVC03, right eye (50 mJ/cm^2^)	Eye discomfort, mild photophobia epiphora sensation	3 h	6 h
UVC04, right eye (75 mJ/cm^2^)	Mild pain, epiphora, conjunctival hyperemia	2 h	11 h
UVC05, right eye (75 mJ/cm^2^)	Eye discomfort, mild pain, epiphora	2 h	10 h

## DISCUSSION

In the present study, dose‐controlled far‐UVC was irradiated directly to the human eye for check safety profile: no clinically identifiable photokeratitis or facial skin erythema was observed after irradiation with 222 nm lamps from 22 to 75 mJ/cm^2^. In rat experiments, the minimal threshold dose of photokeratitis was 5000 mJ/cm^2^; therefore, the present results were well predicted.^13^ However, in the earlier human experiments conducted in the 1970s, photokeratitis was reported to be induced at only 10 mJ/cm^2^.^18^, ^22^ In those previous human studies, by 9 h after the exposures of 215–225 nm UVC, corneal epithelial debris and an increase in corneal light scattering were induced by 3.6 or 5.5 mJ/cm^2^ exposure, and corneal epithelial debris, haze, and granulation were observed, along with a decrease in visual acuity.^18^, ^22^ In these experiments by Pitts, a 5000 W xenon‐mercury high pressure lamp monochromator was used; and because of its small throughput, his experiments were done with very wide (10 nm full width at half maximum) monochromator bands, which therefore introduced large uncertainties created by the large bandwidth and stray‐light (out‐of‐pass‐band) spectral radiant energy.^5^ In this study, the eyes of the subjects were carefully observed by ophthalmologists using slit lamps and other examination instruments. Considering the results of this study, the threshold for clinically detectable photokeratitis is likely to be higher than 75 mJ/cm^2^ as long as well‐filtered lamps that currently can be used for UVGI are employed.

No photokeratitis was observed after the first day of irradiation (i.e., 24 h after the start of irradiation). However, all five subjects reported subjective symptoms that started during or immediately after irradiation, although they did not report visual acuity reduction. These were all symptoms in the right eye and were not reported in the left eye wearing the filter spectacles. Therefore, these symptoms are considered to be a direct effect of UVC irradiation. The human corneal epithelium has five to seven cell layers and an accepted central thickness of approximately 50 to 52 μm^23^, ^24^; this thickness is approximately one tenth of the total corneal thickness. In rat experiments, 222 nm UVC was only able to penetrate one cell layer of the topmost surface layer of the corneal epithelium^13^ and no UV penetration was observed into the germinative cells in the base of the corneal limbus.^14^ This is well explained by the other report that the irradiance of far‐UVC such as 207 nm radiant energy was reduced by half in about 0.3 μm of tissue.^7^ The corneal epithelium is a highly active, self‐renewing layer; a complete turnover occurs in approximately 5 to 7 days.^25^ The typical lifetime of corneal surface cells is about 48 h, so they serve as **“**sacrificial**”** surface cells soon to be sloughed off in the normal corneal epithelial life cycle to act as a protective shield for the underlying corneal epithelium.^5^ Human tear fluid transmits 222 nm UVCs by 90%,^26^ although the data were obtained from collected tear fluid and not from the normal tear film. We therefore believe that the influence of the tear fluid was minimal. Corneal pain is perceived by the trigeminal nerve, whose terminals are distributed in the corneal epithelial cell layer as intraepithelial terminals.^27^ Therefore, some findings, at least findings of conjunctival hyperemia and/or increase in tear meniscus height, could have been observed if observations were made before 24 h. In rat experiments, cyclobutane pyrimidine dimer formation was observed in the most superficial layer of the corneal epithelium within a few minutes after the start of 222 nm irradiation.^13^ However, this change was not accompanied by an obvious loss of corneal epithelium. As corneal fluorescein staining is a test to detect areas of epithelial defects, it is possible that corneal erosions would not have been detected if the test was performed when the patient was symptomatic. This point will be clarified in another clinical study currently planned.

As UV damage to the eyes and skin is known to be fluence‐dependent,^13^, ^28^ eye and skin safety, including ACGIH TLVs, are set at total irradiation dose. In mannequin studies, it has been calculated that in full‐room UVGI settings, the amount of UV radiation incident on the eyes is about 5.8% of the UV dose set for the space.^29^ In the real world, the irradiance incident on the eye may vary further because people move. The effects of short periods of UV irradiation should also be clarified in such situations; additional studies on irradiance‐dependent eye and skin damage are warranted, as little is known about it.

In conclusion, direct irradiation of the eyes with 222 nm far‐UVC up to 75 mJ/cm^2^ delivered at an irradiance of 3.1–3.4 μW/cm^2^ does not induce “clinically significant photokeratitis,” such as that associated with SPK, nor long‐term eye and skin damage. However, it may induce ocular discomfort and tearing sensations that disappear after a short period of time.

## CONFLICT OF INTEREST STATEMENT

This study was conducted as contract research funded by Ushio Inc. to Shimane University. The equipment used for irradiation was provided by Ushio Inc. MT and SK were supported by an unrestricted research donation from Ushio Inc. MS is an employee of Ushio Inc.
